# PRObiotics and SYNbiotics to improve gut health and growth in infants in western Kenya (PROSYNK Trial): study protocol for a 4-arm, open-label, randomised, controlled trial

**DOI:** 10.1186/s13063-022-06211-1

**Published:** 2022-04-11

**Authors:** Mary Iwaret Otiti, Simon Kariuki, Duolao Wang, Lindsay J. Hall, Feiko O. Ter Kuile, Stephen Allen

**Affiliations:** 1 Malaria Branch, Kenya Medical Research Institute (KEMRI) Centre for Global Health Research, P.O. Box 1578, Kisumu, 40100 Kenya; 2 Global Health Trials Unit, Department of Clinical Sciences, Liverpool School of Tropical Medicine, Pembroke Place, Room M-214, Liverpool, L3 5QU UK; 3 Quadram Institute Bioscience, Norwich Research Park, Norwich, NR4 7UQ UK; 4 School of Life Science, Technical University of Munich, Freising, Germany

**Keywords:** Environmental enteric dysfunction, Probiotic, Synbiotic, Inflammation, Gut health, Nutrition, Growth

## Abstract

**Background:**

Malnutrition amongst under-fives remains common in resource-poor countries and is resistant to current interventions. New opportunities have emerged to target “environmental enteric dysfunction” (EED) that refers to the abnormal gut structure and function that results from colonisation of the gut with pathogenic microbes and compromises nutrition and growth in early life. Although the gut microbiome may provide a defence against ingested gut pathogens through colonisation resistance, its development is adversely affected by multiple environmental factors. Dietary supplements of pro- or synbiotics may build the resilience of the gut microbiome against these environmental factors and boost colonisation resistance. We aim to assess whether dietary supplementation of newborns in rural Kenya with pro/synbiotics prevents or ameliorates EED and improves growth.

**Methods:**

Six hundred newborns less than 4 days old will be recruited from Homa Bay County Teaching and Referral Hospital, western Kenya. Newborns will be randomly allocated, stratified by HIV exposure, in a 1:1:1:1 ratio to one of 4 study arms to receive either of two synbiotics, a probiotic or no supplement. Supplements will be given daily for 10 days and then weekly until 6 months of age. Participants will be followed until the age of 2 years. The primary outcome is systemic inflammation at 6 months assessed by plasma alpha-1-acid glycoprotein. Secondary outcomes include biomarkers of gut health and growth, anthropometric indices, morbidity and mortality.

**Discussion:**

As dietary supplements with pro- or synbiotics may improve gut health and can be administered in early life, our findings may inform the package of interventions to prevent malnutrition and improve growth in Africa and similar low-resource settings.

**Trial registration:**

Pan African Clinical Trials Registry, Trial number: PACTR202003893276712. Date: 02/03/2020 https://pactr.samrc.ac.za/TrialDisplay.aspx?TrialID=9798

**Supplementary Information:**

The online version contains supplementary material available at 10.1186/s13063-022-06211-1.

## Administrative information

Note: the numbers in curly brackets in this protocol refer to SPIRIT checklist item numbers. The order of the items has been modified to group similar items (see http://www.equator-network.org/reporting-guidelines/spirit-2013-statement-defining-standard-protocol-items-for-clinical-trials/).

**Table:** 

Title {1}	Gut Power: Protecting the early life gut microbiome to prevent malnutrition: Study protocol of a 4-arm, open-label, individually randomised, controlled, phase-2, study of a probiotic and two synbiotics in young children in western Kenya: ACRONYM: PRObiotics and SYNbiotics in infants in Kenya; the PROSYNK study
Trial registration {2a and 2b}.	Pan African Clinical Trials Registry, Trial number: PACTR202003893276712 Registered in accordance with WHO and ICMJE standards https://pactr.samrc.ac.za/TrialDisplay.aspx?TrialID=9798
Protocol version {3}	Version 7.0, May 3^rd^ 2021
Funding {4}	Children's Investment Fund Foundation
Author details {5a}	Kenya Medical Research Institute - Centre for Global Health Research (KEMRI-CGHR), P.O.Box 1578, 40100 Kisumu, Kenya Department of Clinical Sciences, Liverpool school of tropical medicine (LSTM), Liverpool, UK Quadram Institute Bioscience,Norwich Research Park, NR4 7UA, UK Intestinal Microbiome, School of Life Science, Technical University of Munich, Freising, Germany
Name and contact information for the trial sponsor {5b}	Denise Watson, Research Governance & Integrity Manager, Liverpool School of Tropical Medicine (LSTM) Pembroke Place, Liverpool L3 5QA, UK Phone: + 44 0151 7053100; Email: lstmgov@lstmed.ac.uk
Role of sponsor {5c}	The study sponsor and funder had no role in the design of this trial and will not have any during the execution, analysis, interpretation of the data, or decision to submit the results.

## Introduction

### Background and rationale {6a}

In 2020, more than 1 in 5 under-fives (149.2 million worldwide) were stunted and wasting affected 6.7% of children (45.4 million), of whom 13.6 million had severe wasting [1]. Chronic malnutrition, evidenced by stunting, is associated with significantly impaired cognitive development and the growth and development of other organs [2–4]. An aggregate measure of undernutrition was estimated to account for up to 3.1 m under-five deaths (45% of all child deaths) in 2011 [5].

Current interventions, including exclusive breastfeeding (EBF), have limited impact on growth as achieving 90% coverage with the ten best evidence-based nutrition-specific interventions would reduce stunting by only 20% [6]. In the recent water, sanitation and handwashing (WASH) Benefits studies in Bangladesh and Kenya, children who received food supplements with or without other interventions had improved linear growth but the benefit was limited: in Bangladesh, the mean difference in length-for-age *Z* score at age 2 years was 0.25 (95% confidence interval (CI) 0·15–0.36) in children receiving a nutritional supplement [7] and in Kenya 0.16 [0·05–0·27] in children in the combined water, sanitation, handwashing and nutrition group [8]. Although EBF is universally accepted as the optimal mode of feeding young infants [9], research studies have not found that EBF improves infant growth [10–14].

Targeting “environmental enteric dysfunction” (EED) may offer new opportunities. EED refers to the abnormal gut structure and function that is universal in people living with poor sanitation and hygiene with onset as early as age 12 weeks despite EBF [15]. The gut pathology is characterised by villous atrophy, chronic inflammation and a “leaky” mucosa. The consequences are reduced absorption of nutrients, increased systemic inflammation and impaired immune responses to oral vaccines [15, 16].

Several recent studies have confirmed that, in the context of poor nutrition and in addition to diarrhoeal episodes, frequent sub-clinical enteric infection with organisms that damage the intestinal mucosa results in EED and contributes to systemic inflammation that directly impairs linear growth through decreased production of growth hormones [17–20]. In the MAL-ED study, gut inflammation was most closely associated with poor weight gain whilst systemic inflammation correlated closely with stunting [19]. EED is a sub-clinical condition manifest only by growth failure. Although intestinal biopsy to directly evaluate enteropathy is not feasible or justified in community-based studies [21], the association of multiple biomarkers reflecting the different pathologies of EED and its consequences with growth faltering in children have been assessed [19, 21–23].

### Justification for the study

The gut microbiome is critical for the development of the gut, brain and other organs, mucosal and systemic immunity and protection against gut infection through colonisation resistance [24–27]. In healthy, breastfed infants, bifidobacteria flourish and become abundant. Bifidobacteria facilitate further gut colonisation by a diverse range of anaerobes by “cross-feeding” through hydrolysis of complex carbohydrates and the production of organic acids. Some strains of lactobacilli and bifidobacteria have direct immune-modulatory, anti-inflammatory and anti-pathogen effects, the latter partly mediated through acidifying the intestinal lumen through increased production of organic acids [24, 28, 29]. As key “pioneer” organisms creating the right environment in the gut for colonisation with a broad range of healthy anaerobic bacteria, lactobacilli and bifidobacteria may have long-term benefits that persist into adult life reducing the risk of common metabolic diseases as well as improved long-term gut health and immunity [25].

Several common environmental factors may either delay or disturb the development of the gut microbiome resulting in dysbiosis. These include Caesarean section delivery, hospital admission, maternal/infant antibiotics, poor hygiene and sanitation, parasite infection and faulty feeding practices [29–34]. Additionally, maturity at birth has been found to affect microbiota development with those extremely premature infants at risk of delayed microbiota development [35]. Several studies have reported delayed maturation of the gut microbiome and a deficiency of *Bifidobacterium* in children with malnutrition [36].

Administration of pro- or synbiotics may be a feasible, safe and effective means of building the resilience of the developing gut microbiome against environmental factors in early life. Probiotics are live, non-pathogenic microorganisms that, when administered in adequate amounts, confer a health benefit on the host [37]. Commonly used probiotics are strains of lactobacilli and bifidobacteria. Prebiotics are defined as substrates that are selectively utilised by host microorganisms conferring a health benefit [38]. Prebiotics are present in breast milk as human milk oligosaccharides. Oligosaccharides are commonly used prebiotics and increase bifidobacteria and related taxa [38]. Synbiotics combine prebiotics with probiotics.

The feasibility of administering pro- or synbiotics, including during exclusive breast feeding, was confirmed in a large trial in poor rural communities in India. A synbiotic given for 7 days in approximately 2000 newborns (and 2000 controls), nearly all of whom were breastfed, significantly reduced sepsis, pneumonia and skin infections [39]. A previous search of several registries did not identify any on-going studies evaluating prebiotics, probiotics, or synbiotics in preventing of EED or malnutrition.

### Objectives {7}

#### Goal

This project addresses a pragmatic, public health question: in infants exposed to high levels of faecal-oral pathogen transmission, does the administration of pro/synbiotics early in life improve gut health and growth?

#### Primary objective

To determine whether dietary supplementation with a pro- or synbiotic of newborns during the first 0–5 months of life reduces systemic inflammation at age 6 months assessed by plasma alpha-1-acid glycoprotein (AGP).

#### Secondary objectives

To determine:

1. Whether pro/synbiotic administration improves biomarkers of intestinal inflammation and leakiness, reduces stool pH and improves levels of growth hormones at 6 weeks and 3, 6 and 12 months
2. Whether pro/synbiotic administration reduces episodes of illness (e.g. diarrhoea, respiratory and skin infections)
3. Whether pro/synbiotic administration improves gain in weight, length and head size up to age 2 years
4. Feeding and WASH practices and environmental factors that likely influence the development of the gut microbiome

### Trial design {8}

We will undertake an exploratory, individually randomised, 4-arm, parallel-group, open-label, controlled study of a probiotic and two synbiotics in infants 0–5 months of age in western Kenya.

## Methods: participants, interventions and outcomes

### Study setting {9}

The project will be conducted in rural communities in Homa Bay County in western Kenya. In Nyando Division, a largely rural region in previous Nyanza Province, surveys in 2007–2009 of > 1000 children aged 6–35 months reported that 5% of children were wasted (WHZ < − 2 *z* score) and 28% were stunted (HAZ < − 2 *z* score) [40]. Surveys undertaken in 2014/15 of 858 under-fives in 10 villages (1800 households) reported mean (SD) WHZ score was − 0.12 (± 1.1) with 4.8% children wasted and a mean (SD) HAZ score was − 1.2 (± 1.2) with 23.5% stunted [41]. The WASH Benefits study undertaken in rural villages in the western region reported that in control children with a median age of 2.05 years (IQR 1.93–2.16), mean length-for-age *z* score was − 1.54, 31.5% of children were stunted, 9.3% had severe stunting, 9.6% were underweight and 1.4% were wasted [8]. Around 1 in 4 caregivers reported diarrhoea in their children in the past 7 days.

The project will be based in the Homa Bay County Teaching and Referral Hospital, a 280-bed government facility. The hospital has between 250 and 300 deliveries/month with satellite ante-natal care (ANC) clinics in Homa Bay County. Additional back-up sites in the County may also be included should recruitment be lower than expected.

### Eligibility criteria {10}

#### Inclusion criteria

- Singleton newborn aged 1–3 days
- Birthweight (BW) or current weight (if BW not known) ≥ 2000 g
- Well infant who is breastfed and has taken at least one breast feed well
- Lives within the catchment area of the research centre at Homa Bay Hospital
- Informed consent secured from mother/carer
- Infants of HIV-positive women without known immunosuppression will be eligible to join the study.

#### Exclusion criteria

- Multiple pregnancy (e.g. twin/triplets)
- Infant aged 4 days or older
- Suspicion or presence of any acute illness (e.g. fever; receiving treatment with antibiotics)
- Congenital abnormality that might be life-threatening or impair growth
- Infant with potential contraindication to pro/synbiotics (e.g. suspected immune suppression; cardiac abnormality)
- Mother unlikely to stay in study area for the duration of the study
- Any health staff or study staff concerns regarding safety to participate in the trial

### Who will take informed consent? {26a}

Written, informed consent will be obtained in the local vernacular language by Community Interviewers who will be part of the study team. The consent process shall be initiated when women attend ANC in the last trimester of pregnancy or for delivery. Mother/carers whose newborns may meet the eligibility criteria will have the study explained to them by a member of the study team and provided with an information sheet in an appropriate language. If the newborn meets the eligibility criteria, the consent process will follow, with a written consent form provided. A copy of the informed consent document will be given to the participant for their records unless they state that they do not wish to have a copy.

For illiterate mothers/carers, an independent witness will be present during the informed consent process and will sign the consent form, whilst the participant will be asked to indicate consent by thumbprint. The participant may withdraw consent at any time throughout the course of the study, and this will be made clear in the informed consent process.

All mothers/carers will be informed that there is no requirement to join the study and that standard medical care will remain the same regardless of study enrolment.

The mother/carer will be given the option to take the patient information sheet and consent statement home and encouraged to ensure that the child’s father and other significant family members are aware of the study and agreeable for the child to participate. The child’s father may sign the study consent form as the adult providing consent for baby.

### Additional consent provisions for collection and use of participant data and biological specimens {26b}

A request for informed consent to donate any remaining samples for future research, including possible shipment to laboratories at the Liverpool School of Tropical Medicine, Liverpool, UK, for analyses not available in Kenya, will be included in the consent form. This will allow the study to save and store long term any spare stool and blood samples for possible future analyses relevant to gut health and nutrition in early life. These may include nutrient assays in blood (e.g. iron), detection of the probiotic organisms and specific enteropathogens in stool and microbiota analysis.

## Interventions

### Explanation for the choice of comparators {6b}

The selection of probiotics was based on their previous safe use in infants and manufactured according to Good Manufacturing Practice (GMP) and with good shelf life at ambient temperature. The safety of the Lab4b bacteria in newborns was confirmed in a UK study [42]. Lactobacilli and bifidobacteria were administered routinely in about 70% of preterm infants in Germany [43]. A survey reported that Labinic was used routinely in 7 UK neonatal units [44] and several treatment protocols and patient information sheets are available for preterm infants (e.g. http://swneonatalnetwork.co.uk/media/105774/swnn-guideline-probiotics.pdf). Clinical experience of this product in highly vulnerable preterm infants has not raised concerns regarding safety (Prof. Nicholas Embleton; personal communication). The sensitivity of the probiotic strains to antibiotics used for sick infants in Kenya was confirmed (Additional file 1).

The combination of the probiotics with a prebiotic was based on the reduction in neonatal sepsis when a synbiotic (*Lactobacillus plantarum* ATCC strain 202195 in combination with fructooligosaccharide (FOS)) was administered to newborns in Odisha, India [39]. A neonatal study reported that the BENEO Orafti Synergy 1 prebiotic added to infant formula resulted in comparable *Bifidobacterium* levels in stools compared to breastfed infants and enhanced when compared with GOS:FOS 90:10 supplemented infant formula [45].

The effects of the administration of a probiotic alone versus a synbiotic and the different synbiotic preparations will be assessed by comparing outcomes in the following four study arms.

### Intervention description {11a}

Participants will be randomly allocated to one of 4 study arms:

- Arm 1: Labinic synbiotic daily for 10 days and then weekly to age 6 months
- Arm 2: Lab4b probiotic daily for 10 days and then weekly to age 6 months
- Arm 3: Lab4b synbiotic daily for 10 days and then weekly to age 6 months
- Arm 4: Control group; no dietary supplement

Infants in the control arm will receive the same home visits as those receiving a pro/synbiotic.

#### Details of pro/synbiotics

- Labinic synbiotic: prebiotic (BENEO Orafti Synergy1; 50% oligofructose:50% FOS; 200 mg) + 3 live bacteria: *Lactobacillus acidophilus* NCFM, *Bifidobacterium infantis* Bi-26 and *Bifidobacterium bifidum* Bb-06; total of 5 billion organisms/day (https://biofloratech.com/EU_page_Labinic_Drops.html).
- Lab4b probiotic: 4 live bacteria: *Lactobacillus salivarius* CUL61, *Lactobacillus paracasei* CUL08, *Bifidobacterium animalis subspecies lactis* CUL34 and *Bifidobacterium bifidum* CUL20; total of 10^10^ organisms/day (https://www.lab4probiotics.co.uk/).
- Lab4b synbiotic: prebiotic (long-chain FOS 150 mg/day) + Lab4b probiotic.

All three supplements will be presented as powder inside capsules with one dose/capsule. In this open-label study, the colour of the capsules differs for each preparation to facilitate correct administration. The contents of the capsule can be sprinkled directly into the infant’s open mouth before feeding or mixed in a clean container with sterile water. The administration will be repeated once if the infant vomits within 30 min. All administration of supplements will be supervised by a member of the research team.

In infants taking antibiotics, including HIV-exposed infants taking co-trimoxazole daily from age 4–6 weeks, the supplements will be administered at least 4 h before/after the antibiotic or between doses to minimise the effect of the antibiotic on the microbes. We aim to start the interventions in infants within the first 3 days of birth to limit potential competition from other gut organisms against the probiotics.

Although the dietary supplements are stable at ambient temperatures below 25 °C for up to 2 years, viability at higher temperatures has not been assessed. Therefore, a cold chain was maintained during shipment of the supplements with storage at 5 °C ± 3 °C in Kenya and transfer to participants’ home in a cold box. Quantitative counts of the probiotics will be done at intervals during the study to confirm viability.

### Criteria for discontinuing or modifying allocated interventions {11b}

The dietary supplements will be discontinued in any infants in whom there is a concern by a member of the study team or a health professional that they may be causing harm. Mothers/carers can withdraw their infant from the study at any time should they have any similar concerns. Administration of the pro/synbiotics will be discontinued if there are concerns regarding loss of viability.

### Strategies to improve adherence to interventions {11c}

Mothers/carers will be assisted in administering the dietary supplements to their infants. All administrations will be done under the supervision of study staff who will record adherence. Where feasible, study participants will be reminded about follow-up visits through mobile phone contact. Home visits to both intervention and control infants will include advice regarding healthy feeding and childcare practices.

### Relevant concomitant care permitted or prohibited during the trial {11d}

All participants will be offered routine care according to local and national policies. This includes all childhood vaccinations during infancy and advice regarding the importance of exclusive breast feeding, complementary feeding from age 6 months and sleeping under an insecticide-treated bed net. The study team will re-enforce these positive health messages where appropriate. All routine medications used for the treatment of childhood illnesses is permitted and concomitant medications taken during the study will be recorded with indications and dates of administration. No medications are prohibited. Study staff will facilitate referral to health services for infants who are unwell during scheduled and unscheduled study visits.

### Provisions for post-trial care {30}

The study is not able to fund post-study care or implementation of pro/synbiotics as policy. However, the investigators will ensure that policymakers (e.g. WHO) and funders (e.g. CIFF) are informed early of germane research findings.

### Outcomes {12}

#### Primary outcome

The primary outcome will be raised plasma AGP concentration (> 1 g/L) at age 6 months as a key biomarker of poor gut health contributing to chronic systemic inflammation and impaired growth.

#### Secondary outcomes

Biomarkers at ages 6 weeks and 3, 6 and 12 months

1. Plasma marker of chronic inflammation: (AGP; at 6 weeks and 3 and 12 months)
2. Stool biomarker of intestinal inflammation: myeloperoxidase
3. Stool biomarker of intestinal permeability: α1-antitrypsin (AAT)
4. Plasma marker of acute inflammation: C-reactive protein (CRP)
5. Plasma marker of gut mucosal integrity: intestinal fatty acid-binding protein (IFABP)
6. Plasma growth hormones: insulin-like growth factor (IGF)-1 and its carrier protein IGF-binding protein 3
7. Stool pH

Clinical outcomes to age 2 years

1. Mortality
2. Hospital admission
3. All-cause sick-child clinic visits
4. Disease-specific sick-child clinic visits (e.g. diarrhoea; respiratory and skin infections)
5. Anthropometric outcomes: weight, length, mid-upper arm circumference (MUAC), head circumference

Feeding and WASH practices and environmental factors that may influence the development of the gut microbiome will be assessed by questionnaires.

### Participant timeline {13}

Pregnant women and families will be provided with information about the study when attending ANC during the third trimester. Following the delivery of a live infant, newborns will be assessed by study staff according to the inclusion/exclusion criteria within the first 3 days after birth. After securing informed consent, newborns will be allocated randomly to one of the 4 study arms. Study staff will undertake a Ballard assessment to determine gestation, measure anthropometric indices and record infant feeding, WASH practices and environmental information using standardised questionnaires. Study staff will confirm that the infant has taken at least one breastfeed well and then assist the mother/carer in administering the first dose of the appropriate dietary supplement in infants allocated to the active study arms.

**Fig. 1:**
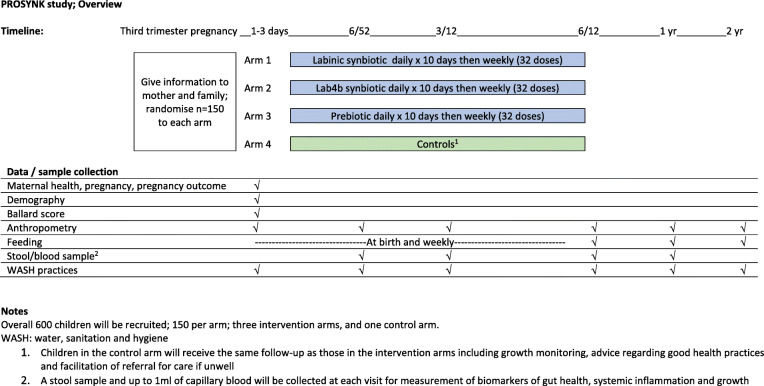
PROSYNK study timeline

Study staff will visit daily for the following 9 days and then weekly to age 6 months to supervise the administration of the pro/synbiotic to infants in the intervention arms. The same visit schedule will be applied to infants in the control arm (no dietary supplement). At all visits, research staff will encourage exclusive breastfeeding and other positive health practices and facilitate transport to the clinic as required in unwell infants. Follow-up visits for all participants to collect data and stool and blood samples will be scheduled for 6 weeks, 3 months, 6 months and 12 months. A final follow-up visit at 2 years will be scheduled to record long-term outcomes (Fig. 1).

### Sample size {14}

A total of 600 newborns will be recruited. For the primary outcome, we predict raised AGP (> 1 g/L) in 35% infants at age 6 months in the control group [22]. The study requires a total of 524 infants (131 per am) to demonstrate a 50% reduction (relative risk = 0.50) in the prevalence of the primary outcome from 35% in the control arm to 17.5% in any of the intervention’s arms with 80% power and *α* = 0.0167. The alpha of 0.0167 is required to allow for three comparisons, one for each intervention arm, against the control arm. We will recruit 150 infants per group to allow for 13% dropouts.

We have used AGP concentration as a dichotomous outcome as a conservative approach as we wish to recruit sufficient numbers of children to compare other important biomarkers between groups. Using values of biomarkers as continuous variables will increase the power of the study to detect differences between the study arms. Using AGP as an example, we predict a mean log_10_ value of − 0.24 g/L in controls based on data from Zimbabwe [20]. In total, 102 infants in the intervention and the control group are required to show that mean AGP concentration is 20% lower in the intervention compared with the control group with 90% power at the 5% significance level.

### Recruitment {15}

Recruitment of 600 newborns is expected to require 12 months with a recruitment rate of approximately 50 newborns per month at the Homa Bay hospital. We plan to pace recruitment by including approximately 12 eligible infants/week to ensure adequate staff and transport are available to complete subsequent follow-up visits. We will recruit infants consecutively according to eligibility but pause recruitment once the target number of infants for that day/week has been reached.

## Assignment of interventions: allocation

### Sequence generation {16a}

The trial statistician at LSTM, UK, will prepare computer-generated random allocation sequences using blocks of random size and stratified according to HIV exposure. The allocation ratio will be 1:1:1:1.

### Concealment mechanism {16b}

The randomisation sequences will use dummy codes (e.g. A, B, C and D). The random sequences will be held by the independent statistician and the trial pharmacist and concealed from all other members of the research team. Another independent statistician who is not involved in the study will assign the dummy codes to each of the four study arms.

### Implementation {16c}

The allocation sequences will be forwarded to the trial pharmacist based in Kenya who will prepare sequentially numbered, sealed, opaque envelopes each with a unique study number according to the random allocation sequences. The envelope will contain a card indicating the intervention group and colour coded to match the supplement capsules. These opaque envelopes will be opened sequentially upon enrolment of each newborn in the presence of the mother/carer and research staff.

## Assignment of interventions: Blinding

### Who will be blinded {17a}

Staff allocating newborns to the study arms and laboratory staff doing the assays for the primary and secondary outcomes will be blinded to the allocation sequence. The trial statistician will also be blinded regarding the treatment code when developing the statistical analysis plan and writing the statistical programmes, which will be validated and completed using dummy randomisation codes. The allocation sequence will only be provided to the study team after locking of the database for the primary outcome, and after the statistical analysis plan has been signed off by the Chief Investigator, the trial statistician and a representative of the independent Data Monitoring and Ethics Committee (DMEC).

### Procedure for unblinding if needed {17b}

This is not required in this open study.

## Data collection and management

### Plans for assessment and collection of outcomes {18a}

Biomarkers of gut health and growth will be measured in capillary blood samples (up to 1 ml) collected during visits to the research clinic and stool samples collected from homes at 6 weeks, 3 months, 6 months and 12 months. The stool samples obtained from homes will be collected by the mother/carer who will have been provided with materials to obtain a late evening or early morning stool prior to going to the facility for the scheduled visit. The materials provided include a disposable nappy or plastic sheet, pre-labelled (with participant ID, date and visit) sample container with a scoop, plastic specimen bag, small cold box with 3 frozen ice packs with a biohazard label, a bar of soap, gloves, tissue paper, biohazard bag and ziplock bags. The mother/carer is asked to collect stool from the nappy or plastic sheet until 2 ml if the usual consistency is liquid or semi-formed or the size of two marbles if formed. Study staff prior to stool collection will inquire if the child had diarrhoea (described as three or more loose stools in 24 h) in which case stool collection will be suspended until the episode has subsided. The sample will be delivered to the lab within 12 h from the time the icepacks are packed into cooler box. The time from sample collection to receipt in the laboratory will be recorded.

In plasma, AGP (Human alpha 1-Acid Glycoprotein Quantikine ELISA), CRP (Human C-reactive protein/CRP quantikine ELISA) and IFABP (Human FABP2/IFABP Quantikine ELISA), IGF-1 and IGFBP3 (Human IGF-I and IGFBP-3 Quantikine ELISA) in kits provided by fbio-techne.com, Abingdon, UK. In stool, myeloperoxidase (Human Myeloperoxidase Quantikine ELISA; fbio-techne.com, Abingdon, UK) and AAT (Immunodiagnostik AG; Hamburg, Germany) will be measured. Kits from other manufacturers may also be considered. For stool pH, an aliquot of stool will be diluted 1:10 in sterile water, mixed with a vortex mixer for 4 min, centrifuged to precipitate solids (2 min, 12,000 relative centrifugal force) and pH measured in the supernatant using a pH metre [46].

Laboratory assays will be carried out according to the manufacturer’s instructions. All assays will be carried out in duplicate and repeated should there be a discrepancy in values. Processing, analysis and storage of samples will be undertaken at the Research Laboratories at the Kenya Medical Research Institute (KEMRI) Centre for Global Health Research, Kisumu, Kenya.

Clinical outcomes will be assessed as follows:

1. Mortality and hospital admissions will be recorded by a questionnaire applied during each scheduled and unscheduled visit and information copied from any health cards carried by the mother/carer and/or from hospital records and reported as serious adverse events (SAEs).
2. Episodes of all-cause and disease-specific sick-child clinic visits will be recorded during each scheduled visit as reported by the mother/carer and information copied from any health cards carried by the mother/carer and/or from clinic records.
3. Weight will be measured to the nearest 100 g using electronic scales. MUAC will be measured at the mid-point between the tip of the shoulder and the tip of the elbow using a non-stretchable MUAC tape to the nearest 0.1 cm. Length will be measured to the nearest 0.1 cm using a length board. Head circumference will be measured to the nearest 0.1 cm using a non-stretchable tape measure. All measurements will be performed in duplicate and a third measurement made should values disagree by a pre-specified amount. Scales and length boards will be checked daily for accuracy using a standard weight and a ruler.

### Plans to promote participant retention and complete follow-up {18b}

Several community sensitisation meetings were held in Homa Bay town sub-county and approximately 5 km radius before the start of the study to provide information and hear and respond to questions or concerns raised regarding the study. These meetings will continue during the study as forums to raise and address any concerns. During screening, women will be asked whether they live in the catchment area and are willing to adhere to the study protocol including being available for follow-up visits. Detailed directions to participants’ homes as well as contact information, including mobile phone numbers, will be recorded at enrolment. If mothers/carers are not available or present at scheduled follow-up visits, the study team will call them to reschedule the visit or ask them to come to the research clinic at Homa Bay Hospital for evaluation, offering transport reimbursement. Subjects judged to be non-compliant may continue in the study with frequent checks on participant well-being by the study team to address study-related concerns, prevent misinformation from arising and allow continuity with study procedures. Mothers/carers will be provided with a mobile phone number for members of the research team to facilitate communication.

### Data management {19}

Data will be collected on case report forms (CRFs) and questionnaires in paper format. The quality of data collection and data entry will be maximised through training of field staff in standardised methodology and by range and missing data checks during the study. Field staff will be required to demonstrate competence before conducting fieldwork. Forms using HP Teleform software will be designed for semi-automated transcribing of data into an electronic database using scanning and Optical Character Recognition, intelligent document recognition and data validation using checksum algorithms, cross-field validation, range and consistency checks. Verification of information against source documents will also be undertaken by data managers. Once validated, the data will be transferred to the target database along with a PDF of the original image of the CRFs, to generate an electronic copy of all paper-based documents.

Verified and validated data will be stored via the cloud on a secure, highly fault-tolerant, storage area network servers at KEMRI in Kisumu and at LSTM in Liverpool. Data will be backed up in Kenya on a continuous basis on a secure off-site server and on encrypted standalone hard drives. Once the data validation phase is completed by the Data Manager at LSTM, the database will be locked and transferred for statistical analysis at LSTM including further consistency checks and data cleaning (e.g. in Stata). The final cleaned database will be available as SAS, STATA and in SPSS format, with an embedded data dictionary.

The full study protocol, supporting documents and the fully anonymised, individual participant-level database will be made publicly available once the study findings have been published. The Data Manager will produce a document summarising the methods used to generate the data with a full description of all procedures, analyses, data capture tools, coding and description of variables which will be published alongside the research database.

The research data will be stored long term in the original electronic format, in a unified database and a public database that contains all research data other than participant identifiable data. The anonymised data will be preserved for at least 10 years.

### Confidentiality {27}

All information regarding the participants will remain confidential to the extent allowed by law. Unique numerical identifiers will be used for data entry. All screening forms and CRFs will be kept in a secured location with access limited to authorised study staff. Unique numerical identifiers will be used for the computer-based data entry and stool and blood samples. Publications will contain only aggregated data; no identifying information will be included to ensure individual patient anonymisation of all data and results made public.

### Plans for collection, laboratory evaluation and storage of biological specimens for genetic or molecular analysis in this trial/future use {33}

Any remaining stool and blood samples will be stored long term for possible future analyses relevant to gut health and nutrition in early life. These may include nutrient assays in blood (e.g. iron), detection of the probiotic organisms and specific enteropathogens in stool and microbiome analysis. Shipment of the minimum aliquots of stool or serum for analysis in the UK would only be undertaken following approval from the relevant authorities.

## Statistical methods

### Statistical methods for primary and secondary outcomes {20a}

Demographic, clinical and laboratory data and also variables reflecting feeding, WASH practices and environmental factors will be summarised using simple descriptive statistics. Continuous variables will be summarised according to the number of subjects with non-missing data as mean, standard deviation, median, minimum and maximum. Categorical variables will be summarised according to the absolute frequency and percentage of subjects in each category level. The denominator for the percentages is the number of subjects with data available unless noted otherwise.

The primary outcome analysis will be based on the intention-to-treat (ITT) population which will include all randomised study subjects. The proportion of infants aged 6 months with abnormal AGP concentration (> 1 g/L) in each intervention arm will be compared with the proportion in the control arm. Data will be analysed using generalised linear models with treatment as the only predictor, AGP concentration at baseline as covariate, binomial distribution and log link function, generating the estimates of treatment effects in relative risk and their 95% confidence intervals (CI). Covariate-adjusted analysis will also be performed with covariates that affect the primary outcome (e.g. season of birth, HIV exposure, mode of delivery, mode of feeding, exposure to antibiotics, WASH and environmental factors such as water source, sanitation facilities, hygiene practices, use of soap, number of siblings, proximity to animals). Subgroup analysis will also be performed on the covariates used in the covariate-adjusted analysis.

Analysis of the secondary endpoints with single measurements will also be presented in the ITT and per-protocol (PP) populations and in a similar fashion as for the primary endpoint analysis using generalised linear method. The PP population will be all randomised study subjects who complete 75% or more of the supervised doses. For continuous outcomes, normal distribution and identity link function will be used, from which mean difference together with its 95%CI will be derived. Generalised linear mixed model (GLMMIX) will be employed for the analysis of secondary outcomes with repeated measurements. The GLMMIX model will have treatment, visit, interaction between treatment and visit as fixed effects, baseline measurement of an outcome as covariate, and subject as random effect. The treatment difference between two treatment arms at each visit together with its 95% confidence interval will be derived from the GLMMIX model. Missing data will be treated as missing completely at random in the GLMMIX model analysis and no imputation of primary or secondary endpoints will be made. For the analysis of binary secondary outcomes with repeated measurements, the GLMMIX model will have a binomial distribution and logit link function. The odds ratio between two treatment arms at each visit together with its 95% confidence interval will be derived from the GLMMIX model. For the analysis of continuous secondary outcomes with repeated measurements, the GLMMIX model will have a normal distribution and identify link function. The mean difference between two treatment arms at each visit together with its 95% confidence interval will be derived from the GLMMIX model.

For anthropometric outcomes, *z* scores will be calculated using the WHO Anthro Survey Analyser and WHO 2006 child growth standards [47]. Analyses will include both the comparison of anthropometric indices between intervention groups at different time points and also growth expressed as change in indices over time.

Analyses of the potential adverse effects of the dietary supplements will be done in the safety population.

### Interim analyses {21b}

No interim analysis is planned.

### Methods for additional analyses (e.g. subgroup analyses) {20b}

Additional analysis in the ITT population and the per-protocol (PP) population will include important sub-groups such as infants exposed to HIV infection and those delivered by Caesarean section. Additional analyses may be considered should any new information become available.

### Methods in analysis to handle protocol non-adherence and any statistical methods to handle missing data {20c}

Analyses of the PP population will be a sensitivity analysis to investigate whether conclusions are sensitive to assumptions regarding compliance with the dietary supplements and missing data. Maximum efforts will be made to avoid missing values; however, where this does occur, missing data will be reported and left out of analyses.

### Plans to give access to the full protocol, participant-level data and statistical code {31c}

The full study protocol will be available upon request. Requests to access the fully anonymised, raw data would be reviewed by the investigators based at LSTM and Kenya (or their representatives if appropriate). Approval to access the data will be granted only if the request is approved by all of the investigators. The KEMRI SERU and LSTM Research Ethics Committees will be notified of any agreements to share data.

## Oversight and monitoring

### Composition of the coordinating centre and trial steering committee {5d}

The Trial Management Group (TMG) is responsible for the administrative management and day to day running of the trial. It is composed of the Trial Manager, lead Data Manager, lead administrator, Chief Investigator (ad hoc) and other staff who are involved in the day to day running of the trial are invited ad hoc. The main roles are study planning, organisation of Trial Steering Committee (TSC) and Data Monitoring Committee (DMEC) meetings, providing risk report to regulators, manufacturers and ethics committees, serious unexpected suspected adverse events reporting, maintaining the trial master file, budget administration and contractual issues, advice for lead investigators and organisation of central data management and sample collection.

The TSC will meet annually and is composed of an independent chair, two other independent members, a mother/carer representative, a sponsor representative and the funder’s (CIFF) representative who are invited to attend as observers. The Chief Investigator, Trial Statistician, Trial Manager and Programme coordinator are also members. As the trial governing body, the TSC concentrates on the progress of the trial and ensures that the trial is conducted to the standards set out in the Guidelines for Good Clinical Practice with priority given to participant safety. In its first meeting, the nominated members of the DMEC were approved. The TSC provides a summary report and recommendations which are submitted to the funder, the sponsor and the TMG.

### Composition of the DMEC, its role and reporting structure {21a}

Independent members of the DMEC consist of the chair, a topic expert/paediatrician and a statistician, all with experience in clinical trials. The DMEC will convene either face-to-face or remotely twice during the period of recruitment to the study and then annually (before the annual TSC meeting) during the period of follow-up. The members will review results according to masked study arm. Their priority is participant safety and preventing participants from being exposed to any excess risks by recommending to the TSC for trial suspension or early termination early if the safety or efficacy results are sufficiently convincing. The trial statistician is usually invited to attend part of the DMEC meeting to present the most current data from the trial.

### Adverse event reporting and harms {22}

The safety of the infants participating in this study is foremost. Although we consider that none of the study procedures pose a significant risk to the participating infants, the principles of ICH GCP require that both investigators and sponsors follow specific procedures when notifying and reporting adverse events (AEs) or reactions in clinical trials. Research staff will maintain close contact with mothers/carers including providing contact phone numbers. We will document episodes of illness both during study visits to homes and by monitoring attendances at the health facilities where the research is based and continue this to age 2 years.

Although the pro- and synbiotics used in this study are classified as food supplements, we will take a conservative approach and apply the standard definitions applicable to investigational medicinal products. Infants who develop AEs will be identified by parents/carers contacting study staff or at follow-up visits. The intensity of each AE recorded in the case report form will be assigned to a grade (1-5), which will be determined following the definitions set forth in the Common Terminology Criteria for Adverse Events v3.0 (CTCAE) (Cancer Therapy Evaluation Program, 2006). If appropriate, participants will be referred to the clinic or hospital for evaluation and treatment according to local guidelines. Mild AEs will be noted in the participant’s CRF; no further action will be taken by study staff except in the case of vomiting when the pro/synbiotic may need to be re-administered. In the case of a serious adverse event (SAE), subjects will be referred to the hospital for appropriate assessment and management and transportation costs provided. All participants with SAEs will undergo a clinical record review to identify potential adverse consequences of study participation.

The responsible study clinician will use clinical judgment to assess the relationship between the administration of the supplements and the occurrence of each AE/SAE. Alternative causes, such as the natural history of an underlying disease, concomitant therapy, other risk factors, and the temporal relationship of the event to the investigational product will be considered and investigated. The investigator will also consult the product information and the DMEC as needed in the determination of his/her assessment. There may be minimal information to include in an initial SAE report; the investigator may change his/her opinion of causality in light of follow-up information, amending the SAE case report form accordingly.

All SAEs will be reported to the in-country principal investigator or an assigned representative within 24 h of the research staff becoming aware of it, using an SAE form sent electronically. The SAE form documents the nature of the event, date of onset, severity, corrective therapies given, outcome and causality (i.e. unrelated, unlikely, possible, probably, definitely) determined by the responsible study clinician. SAEs that are unexpected and are at least ‘possibly related’ to the study intervention require expedited reporting within 24 h of the in-country principal investigator or assigned representative becoming aware of it (e-mail notification). This will be a maximum of 48 h after the event occurred or the study team were made aware of the event (including the 24 h required for the field staff to report to the principal investigator/representative). Additional information will be sent within 14 additional days (full SAE report) if the event had not resolved at the time of e-mail notification.

Other SAEs and AEs will be reported annually (or more frequently if required by the DMEC or ethics committees) in an aggregated report. AEs that will not be reported include common illnesses that do not result in hospitalisation, including but not limited to clinical malaria, respiratory, gastrointestinal and skin diseases, unless they are considered at least possibly related to the intervention.

The study will comply with local regulations pertaining to reporting SAEs to the local Research Ethics Committee and/or Research & regulatory offices, the primary ethics committees, DMEC and the sponsor. A copy of the final study report will be provided to all study hospitals, ethics committees, TSC, DMEC and the Kenya Pharmacy and Poisons Board.

### Frequency and plans for auditing trial conduct {23}

The trial may be audited at any time by a study staff from the sponsor’s at LSTM in Liverpool, UK. At the discretion of the sponsor, the auditor may accompany the clinical monitor during site visits. External clinical trial monitoring visits are provided by the sponsor at trial initiation, and then regularly (at least yearly) thereafter and at trial closeout, or more frequently if so required. KEMRI has its own internal quality control team who, if so required, will conduct internal monitoring regularly, and help prepare for external monitoring visits. In addition, Quality Assurance/Regulatory Officers from a sister study in KEMRI, Kisumu, will conduct internal monitoring of the study according to established practices. Internal monitoring will include a review of study procedures, standard operating procedures, Investigator Site File, laboratory procedures, training logs and adherence to protocol and GCP guidelines.

### Plans for communicating important protocol amendments to relevant parties (e.g. trial participants, ethical committees) {25}

Protocol amendments will be submitted to the research ethics committees at LSTM (sponsor), the KEMRI-SERU and KPPB for approval before implementation. Any change to the informed consent form, with the exception of layout, spelling errors and formatting, must also be approved by the sponsor and the ethics committee, before the revised form is used.

No change will be made to the approved protocol without the agreement of the sponsor. The Chief Investigator, or a delegated person, will distribute amendments on behalf of the sponsor to the principal investigator, who in turn is responsible for the distribution of these documents to the staff at his/her study site and appropriate staff training.

### Dissemination plans {31a}

This project will generate new information about the infant’s health and well-being, feeding practices and environmental exposures in western Kenya with direct relevance to similar settings. We will liaise closely with government, charitable and private health staff to share information to maximise benefits to the local population. This will be facilitated through our existing links with the Kenyan Ministry of Health, Kenya Paediatric Association, UNICEF, WHO and Commonwealth Society of Paediatric Gastroenterology, Hepatology and Nutrition. In addition, we will provide direct feedback to the County and Sub-County Health Management Teams in HomaBay County, who will facilitate discussions with the community regarding the findings.

We will share our findings with the many researchers working in malnutrition, gut health, the microbiome and pro/synbiotics through presentations at national and international conferences and open-access publications in the scientific and lay press. This project complements other projects in gut health in early life that we are either already undertaking or planning in West/Central Africa, Asia and the Pacific region and we will also use these networks to disseminate our findings. Authorship of scientific reports will follow international criteria. We do not intend to use professional writers.

## Discussion

In this study, we will test whether multi-strain, high-dose preparations of live bifidobacteria and lactobacilli either alone or combined with a prebiotic improve gut health in young infants exposed to poor sanitation and hygiene and at risk of growth faltering.

The choice of pro/synbiotics to evaluate in intervention studies is challenging for several reasons. For many intestinal conditions, including EED, there are multiple abnormalities in intestinal structure and function and, therefore, multiple ways in which an intervention may be beneficial. Equally, pro- and synbiotics may improve health through several different mechanisms, ranging from colonisation resistance, improving intestinal integrity and barrier function, improved nutrient digestion and absorption, and stimulation of mucosal and systemic immunity. The specific properties of pro- and synbiotics in vivo are incompletely understood illustrating the challenge of matching a specific pro/synbiotic preparation to a specific intestinal disorder. Further limitations are the availability of products that are manufactured to GMP and have an established record of safe administration to specific patient groups. Factors that require consideration are single versus multi-strain probiotics, the ability of the selected probiotic(s) to metabolise HMOs in the case of breast-fed infants and the prebiotic in a synbiotic preparation and to persist in the gut long term. Each of these issues requires further research.

Although probiotics have a well-established safety record including in highly vulnerable populations such as preterm infants [48], some adverse events have been noted in specific contexts such as preterm infants with indwelling catheters and receiving glucose infusions that may promote biofilm formation by *L. rhamnosus* sp. [49] and children receiving intensive care [50]. Although the probiotics used in this study have an established safety record in infants and we will recruit only well newborns with no health concerns, this is the first time to our knowledge that these specific probiotics formulated as synbiotics have been used in this setting. Therefore, we have established robust methods for the reporting and review of AEs and also optimised communication with study mothers and families to support the health of their infants.

Pragmatic considerations are also critical for an intervention that may be used a scale and in poorer rural or peri-urban communities. Many probiotic preparations remain viable long term at ambient temperatures in cooler climates but this needs to be determined in tropical zones. In our study, we decided to maintain a cold chain to optimise viability and will also check viability at the point of use throughout the study.

It is also worth noting that the trial is being conducted in the middle of the COVID-19 pandemic. Despite the challenges of possible transmission, the study procedures rely on face-to-face interactions. The study plans to continue with face-to-face data collection activities, with strict compliance with the Ministry of Health (MoH) guidelines on COVID-19 prevention [51–53]. In addition, the study setting faces frequent health care worker unrest that makes it challenging to conduct study activities during the period of unrest. However, with support from the Homabay County government, Ministry of Health and access to additional health facilities, the study has put plans in place to mitigate the negative effects of the strikes to maintain the integrity of the study activities and well-being of study participants.

Overall, modulating the developing gut microbiome in young infants may offer an additional intervention to improve gut health, nutrition and growth. The head-to-head comparison of three different pro/synbiotic preparations with the measurement of several clinical and laboratory endpoints will inform this promising area of research.

## Trial status

*This protocol is Version 7.0, 3rd May 2021.*

Recruitment is expected to be completed at the end of December 2021. The follow-up to age 6 months of all infants is expected to be completed by June 2022; laboratory and data analysis regarding the main outcomes at age 6 months will be reported by October 2022. Outcomes at age 12 months will be reported by April 2023, and final project outcomes including 2-year follow-up will be reported by April 2024.

## Supplementary Information

**Additional file 1.** Antibiotic sensitivity of probiotic organisms.

## Data Availability

The datasets used and/or analysed during the current study are available from the corresponding author on reasonable request. Requests to access the fully anonymised, raw data would be reviewed by the investigators based at LSTM and Kenya (or their representatives if appropriate). Approval to access the data will be granted only if the request is approved by all of the investigators. The KEMRI SERU and LSTM Research Ethics Committees will be notified of any agreements to share data

## Abbreviations

AAT: α_1_-Antitrypsin
AE: Adverse event
AGP: α_1_-Acid glycoprotein
ANC: Ante-natal care
BW: Birthweight
CI: Confidence interval
CIFF: Children’s Investment Fund Foundation
CRF: Case report form
CRP: C-reactive protein
DMEC: Data Monitoring and Ethics Committee
EBF: Exclusive breastfeeding
EED: Environmental enteric dysfunction
GCP: Good Clinical Practice
GMP: Good Manufacturing Practice
HIV: Human immunodeficiency virus
HMOs: Human milk oligosaccharides
IFABP: Intestinal fatty acid-binding protein
IGF-1: Insulin-like growth factor-1
IGFBP3: Insulin-like growth factor-binding protein 3
ITT: Intention to treat
KEMRI: Kenya Medical Research Institute
KPPB: Kenyan Pharmacy & Poison Board
LSTM: Liverpool School of Tropical Medicine
MUAC: Mid-upper arm circumference
SAE: Serious adverse event
SERU: Scientific and Ethics Review Unit
TMG: Trial Management Group
TSC: Trial Steering Committee
WASH: Water, sanitation and hygiene
WHO: World Health Organization
