# Supplementary material for: PRObiotics and SYNbiotics to improve gut health and growth in infants in western Kenya (PROSYNK Trial): study protocol for a 4-arm, open-label, randomised, controlled trial
Source: Trials. 2022 Apr 11;23:284. doi: 10.1186/s13063-022-06211-1 (PMC8996226; doi:10.1186/s13063-022-06211-1)
Supplement: Supplementary file 1 — Additional file 1. Antibiotic sensitivity of probiotic organisms. [file 13063_2022_6211_MOESM1_ESM.docx]

**Additional file 1: Antibiotic sensitivity of probiotic organisms**

| **Agent** | **ARU* organism identification** | **Minimum inhibitory concentration (mg/L)** | | | | | | | | | | | | **Comment** |
| --- | --- | --- | --- | --- | --- | --- | --- | --- | --- | --- | --- | --- | --- | --- |
|  |  | **AK** | **AMP** | **AML** | **AZM** | **CTX** | **CAZ** | **CRO** | **CIP** | **SXT** | **ERY** | **MTZ** | **PEN** |  |
| **Labinic synbiotic** | *Lactobacillus acidophilus* | 32 | 0.047 | <0.016 | 0.032 | 0.19 | 1 | 0.19 | >32 | >32 | 0.023 | >256 | 0.008 |  |
|  | *Bifidobacterium longum* | >256 | 1 | 0.5 | 0.38 | 3 | 4 | 2 | >32 | - | 0.19 | 4 | 0.25 | *B. infantis* is a subsp of *B. longum* (Mattarelli *et al*. 2008*)*. Unable to differentiate to subspecies level using 16S sequencing or MALDI-TOF MS |
|  | *Bifidobacterium bifidum* | >256 | 0.023 | 0.023 | 0.125 | 0.094 | 0.5 | 0.023 | 4 | 0.25 | 0.064 | 4 | 0.023 |  |
| **Lab4b synbiotic** | *Lactobacillus casei group* | 24 | 2 | 1 | 1 | >32 | >256 | >32 | 1 | >32 | 0.25 | >256 | 0.38 | Unable to differentiate *L. casei/paracasei/rhamnosus* using 16S sequencing. Penicillin MIC 0.38 mg/L = I (Susceptible, increased exposure; EUCAST guideline, v10, 2020) |
|  | *Lactobacillus salivarius* | 24 | 0.5 | 0.25 | 2 | 0.5 | 16 | 0.75 | >32 | >32 | 0.5 | >256 | 0.125 |  |
|  | *Bifidobacterium bifidum* | >256 | 0.032 | 0.032 | 0.19 | 0.5 | 1 | 0.75 | >32 | 0.5 | 0.094 | 4 | 0.023 |  |
|  | *Bifidobacterium animalis* | >256 | 0.125 | 0.094 | 0.19 | 0.5 | 0.75 | 0.5 | 4 | 0.38 | 0.064 | 16 | 0.094 | Organism was identified from an original culture of Lab4b by MALDI-TOF MS. Unable to isolate in pure culture; additional testing was performed on isolate provided by Cultech. Unable to identify to subspecies level. |

*Anaerobe Reference Unit, Public Health Wales Microbiology Cardiff, University Hospital of Wales, Heath Park, Cardiff, CF14 4XW, UK
